# HIV Related Knowledge, HIV Testing Decision-Making, and Perceptions of Alcohol Use as a Risk Factor for HIV among Black and African American Women

**DOI:** 10.3390/ijerph18094535

**Published:** 2021-04-24

**Authors:** Angela Wangari Walter, Cesar Morocho

**Affiliations:** 1 Department of Public Health, Zuckerberg College of Health Sciences, University of Massachusetts Lowell, Lowell, MA 01854, USA; 2 Department of Biomedical Engineering, Francis College of Engineering, University of Massachusetts Lowell, Lowell, MA 01854, USA; Cesar_Morocho@uml.edu

**Keywords:** African American women, alcohol use, Black women, HIV/AIDS, HIV knowledge, HIV testing

## Abstract

The HIV/AIDS epidemic disproportionately affects Black and African American women in the United States. This study examined the extent of HIV related knowledge, HIV testing decision-making, and perceptions of alcohol use as a risk factor for HIV among Black and African American women in urban and suburban communities. Seven focus groups were conducted with 37 women aged 18 to 49 residing in the Commonwealth of Massachusetts. Women participating in focus groups had a wide breadth of HIV related knowledge. Findings suggest the influence of interpersonal relationships and provider–patient interactions on HIV testing, the need for building community capacity and leveraging community resources towards HIV prevention, and the influence of alcohol outlet density on HIV vulnerability and exposure in communities of color. Comprehensive multifaceted evidence informed interventions that are culturally relevant and gender responsive are needed to reduce HIV/AIDS disparities.

## 1. Introduction

Despite advances in HIV prevention, diagnosis, treatment and care support, HIV remains a serious public health problem globally with approximately 1.7 million new HIV cases worldwide in 2018 [1,2]. In the U.S., HIV disproportionately impacts Black and African Americans. In 2018, Black and African Americans made up 13% of the U.S. population, but accounted for three times as many new HIV diagnoses (39%), almost half (47%) of stage 3 (AIDS) classifications, and the highest rate of death for individuals with diagnosed HIV infection (16.3 per 100,000) [1]. Black and African American women bear a disproportionate burden with more than half (58%) of new HIV infections compared to their White (21%) and Latina (17%) counterparts [1]. Heterosexual transmission accounts for the largest proportion of HIV infections (92%) among Black and African American women with the remaining 8% resulting from injection drug use [1]. Research shows that the likelihood of Black and African American women being diagnosed with HIV is significantly higher (1 in 54) compared to their Latina (1 in 256) and White (1 in 941) counterparts [3]. A key challenge in HIV prevention efforts is the lack of awareness of one’s HIV status. Surveillance data show that one in nine women living with HIV are unaware of their status, resulting in HIV transmission, missed opportunities for early diagnosis and initiation of antiretroviral therapy (ART), and linkages to HIV medical care and social supports [1]. In the U.S., HIV testing rates are also lower for women who engage in sexual behaviors that increase the risk of acquiring HIV [1].

Social and structural factors have been associated with adverse health outcomes in socially and economically disadvantaged groups defined by race/ethnicity, geography and gender [4,5]. Social and structural determinants of health including socioeconomic position (i.e., income and wealth, educational attainment, employment and occupational rank), housing, transportation, food access and security, neighborhood characteristics, health care access, racial and gender discrimination, racism and intimate partner violence (IPV) shape HIV exposure and the realities of living with HIV once diagnosed [6,7]. For example, drivers of the disparities in HIV infection among Black and African American women are influenced by socioeconomic status [8,9,10,11], inadequate access to healthcare [11], neighborhood conditions including poverty and residential segregation [12,13,14] and intersecting forms of racial discrimination including inferior discriminatory medical treatment resulting in mistrust of the medical system [11,15]. The disproportionate incarceration rates of Black and African American men and women are also linked to increased HIV exposure [16,17]. The disparities in HIV infection among Black and African American women are inherently markers of systemic social and structural inequities that increase vulnerability for HIV infection and transmission. Once diagnosed with HIV, these social and structural inequities elevate disparities in HIV care and treatment outcomes. Specifically, social and structural barriers prevent women from accessing and accepting HIV care and treatment services [18], and achieving viral suppression and adhering to ART [19]. Additionally, these social and structural factors contribute to suboptimal quality of care and reduce the quality of life for women living with HIV [20,21].

The social stigma of HIV/AIDS, including individual level stigma (internalized, anticipated and enacted), structural stigma (expecting or fearing discrimination and structural inequities) and intersectional stigma (interacting multiple stigmas), can negatively impact health seeking behavior and health outcomes [22,23]. HIV stigma may delay or deter women from accessing prevention and treatment services, and thus contribute to greater susceptibility to HIV infection and transmission, and suboptimal care and support for people living with HIV/AIDS (PLWHA) [24]. Women living with HIV report fears and experiences of HIV-related stigma in health care settings as a hinderance to engagement in HIV care [21]. HIV-related stigma may also hinder one’s ability to disclose their HIV status and prevent women from seeking social support [25,26,27].

Gender is an important determinant of health [28] and influences the contexts in which women live, including gender socialization, gender roles and gender inequity, that account for health seeking behavior and health outcomes [29]. Women’s vulnerability to HIV infection and transmission derives from biological aspects [7,30], social and economic inequities, cultural and legal factors and violence against women [7,31,32]. Gender roles, unbalanced power relations and norms play a role in increasing women’s vulnerability to HIV infection [33,34,35], and may affect a woman’s ability to negotiate safe sex practices such as condom use with their partner(s) [36].

The intersection of violence and HIV among women has also been well documented [37]. IPV, whether physical, psychological and sexual, is an important social determinant of health and contributes to greater vulnerability to HIV infection and worse HIV care outcomes [37,38]. Women who have a history of IPV are more likely to engage in risk-taking behaviors such as injection drug use, unprotected vaginal or anal sex, having sex with a male partner at risk for HIV [39]; have multiple (two or more) sex partners in the past year [40]; and to engage in substance use [40,41]. Moreover, the interactions of substance use, violence and HIV/AIDS, also known as SAVA syndemics [42,43], are linked to worse HIV prevention and treatment outcomes. In addition, substance use and misuse perpetuate IPV through HIV sexual risk-taking behaviors and relationship power imbalances [44,45].

Risky sexual behaviors such as unprotected intercourse, having multiple sex partners and participation in survival and transactional sex are considered important risk factors for the transmission of HIV [1]. Research has also shown that some PLWHA continue to engage in transmission-risk behaviors such as unsafe unprotected intercourse [46,47,48,49]. Poor mental health status including depression is associated with greater HIV risk taking behaviors [50,51] and poor adherence to ART [52,53], making mental health treatment critically important in primary and secondary HIV prevention intervention efforts [54]. Alcohol and drug use are also important predictors of HIV infection and HIV disease progression [55]. Drug use has been associated with increased HIV risk-taking including injection drug use [56]. Sexual and drug networks and the related social dynamics of these networks have been shown to increase HIV exposure among Black and African American women [57]. Alcohol use is also associated with unprotected sexual behaviors [58,59] and having multiple sexual partners in the previous year [60]. Taken together, mental health status and substance use (alcohol and drug use) have an effect on women’s ability to adopt and engage in HIV preventive behaviors and influence HIV transmission.

Health literacy, which involves “access to and skillful use of health-related information to inform and improve health decision-making, behaviors and outcomes” [61] (p. 295), is an important factor in the prevention and treatment of HIV. Health literacy is inextricably linked to socioeconomic status and educational attainment [4] with fewer years of schooling and poverty leading to less than adequate literacy and poor health. Low health literacy, i.e., the limited skill for obtaining and acting in ways to benefit health, has been linked to lower HIV disease related knowledge [62,63] and worse health outcomes for PLWHA [61,64] including among Black and African American women [65,66]. Greater educational attainment can lead to improved health by increasing health knowledge (e.g., understanding of HIV risks and modes of transmission), being better-informed when making health decisions and promoting health seeking and preventive behaviors (e.g., HIV testing, engagement with healthcare system). Within the context of HIV disparities, the social and structural contexts in which Black and African American women live may influence health related knowledge and health seeking behaviors, including why, when and how they seek care. For instance, while Black and African American women are more likely to test for HIV in the past year compared to White women (21% vs. 6%) [67], Black and African Americans test for HIV later in their HIV progression, suggesting missed opportunities for HIV testing [68,69]. The factors that influence HIV testing decision-making among Black and African Americans are poorly understood.

The individual, social and structural determinants described here are fundamental drivers of disparities in HIV infection that are not only implicated in Black and African Americans women’s vulnerability to HIV exposure but may also affect their experience once diagnosed. While prior research has identified factors that contribute to greater vulnerability for HIV acquisition and transmission among women, few qualitative studies have examined risk and protective factors from the perspectives of Black and African American women. To address this gap, this study sought to understand: a) the extent of HIV knowledge; b) HIV testing decision-making (when, why, how); and c) perceptions of alcohol use as a risk factor for HIV from the perspective of Black and African American women residing in urban and suburban communities. Findings from this research can contribute to the development of culturally relevant and gender responsive interventions that reflect the realities of Black and African American women.

### Conceptual Frameworks

This study draws on the Public Health Critical Race Praxis (PHCRP) research approach [70] which applies Critical Race Theory (CRT) principles [71] to understand the intersectionality of race as a social construct and factors that contribute to a disproportionate burden of HIV among Black and African American women. CRT allows for a shift from race as a demographic characteristic to an emphasis on how the racialized experiences of Black and African American women might influence behaviors [70]. Using the PHCRP approach, we use CRT principles in conceptualizing the methods, in that instead of comparing Black and African American women to other racial groups, our study focuses on exploring the attitudes, perceptions, and experiences of Black and African American women. Firstly, we apply CRT race consciousness principles and recognize the complex ancestry of Black and African American racial identities and how these identities shape the lived experiences of Black and African American women. We use Black racial identity to refer to individuals having origins of any of the Black racial groups of Africa including the Caribbean, and South and Latin America, etc., while African American identity refers to Americans of African descent with North American ancestry [72,73,74]. We also recognize that women may have multiple ancestries and racial identities.

Secondly, the semi-structured interview guide uses a CRT lens to understand the experiences of Black and African American women within the context of individual, social and structural determinants that contribute to health inequities and influence HIV protective and risk behaviors. Thirdly, considering the overlapping social and structural issues that influence the experiences of Black and African women, we also use the socioecological framework [75,76] both as a conceptual approach and as a methodology for analysis. We examine the contexts of Black and African American women at the individual, interpersonal, community and societal level, and explain how these contexts influence HIV protective and risk factors. The framework recognizes that health behaviors and their consequences, which put Black and African American women at risk for or protect them from HIV infection and transmission, are influenced by the complex interplay of individual, interpersonal, community and societal factors. This integrative socioecological framework along with the PHCRP approach were used in conceptualizing the study, informed the development of the semi-structured interview guide, and allowed for an in-depth analysis of interrelations between individual and contextual level factors contributing to the disproportionate burden of HIV for Black and African American women.

## 2. Materials and Methods

### 2.1. Study Design and Setting

Data were collected from seven focus groups with women residing in the Commonwealth of Massachusetts between January 2012–April 2012. The primary source of stratification for the sampling framework was towns and cities representing a demographic profile of over 10% non-Hispanic Black and African Americans in the town/city (community) [77]. This stratification yielded urban (*n* = 4) and suburban (*n* = 3) communities. Communities were delineated as urban if the population density was greater than 100,000 while suburban communities did not have more than 100,000 people [78,79]. Six of the seven communities were among the top 10 towns/cities in Massachusetts with high proportions of Black and African Americans diagnosed with HIV in the last three years (2014–2016) [80]. Women age 18 to 49 were recruited from their natural environments such as community-based organizations, faith-based organizations, community meetings and agencies/organizations providing services to women. Participants were also recruited in door-to-door interactions at beauty parlors/shops, health and wellness centers and public spaces such as libraries. In addition, a key person at each agency/organization facilitated recruitment efforts by posting and distributing the study flyer and informing clients/patrons of the purpose and objective of the study. Participants completed a consent form approved by the Brandeis Committee for Protection of Human Subjects (protocol number 12005) and were compensated for their time with a $25 gift card. Focus groups were conducted in English, audio recorded and required about an hour and a half to complete.

### 2.2. Analysis

A hybrid approach to thematic analysis incorporating inductive [81] and deductive [82] processes was utilized. This approach enabled the analysis to incorporate the socioecological framework for deductive analysis, while allowing for emerging patterns to be coded using an inductive process. Two coders (AWW and CM) independently engaged in data immersion by actively listening to recordings, reviewing field notes, reading transcripts and writing memos that summarized key points.

A data template with broad categories based on the research questions and the socioecological framework was developed for organizing data. Conceptual categories informed by the framework and HIV/AIDS literature were identified. Transcripts and the analytic template of codes were then entered into NVivo 10 software and analyzed [83]. Segments of data were coded and categorized into the preliminary codes based on research domains and the nested layers of the framework. The deductive approach to coding among and within the nodes was done to determine how themes were (or not) related to the overarching conceptual categories and research domains.

The inductive approach allowed for discovery of naturally occurring themes. This process required reviewing each participant’s statement and comparing it to other pieces of data that were similar or different. Recurring and unexpected content that did not fall in the predetermined categories was coded. Coders then made connections between the codes noting similarities and differences between groups and resolving areas of potential conflict. Once thematic saturation was reached, content and parameters of the data codes were refined. Thematic responses were then coded for intensity, frequency and extensiveness [84,85]. A schematic of the coding process is shown in Table 1.

## 3. Results

### 3.1. Sociodemographic Characteristics of the Study Sample

Table 2 summarizes the sociodemographic characteristics of the study sample. We conducted seven focus groups with an average of five participants (SD 2.4) in each group with no notable variations in demographic proportions across and within groups. As shown in Table 2, a large proportion (60%) of participants lived in urban communities. Thirty-seven Black and African American women who averaged 39 (SD 8.15) years of age participated in the study. Almost all participants (97%) self-identified as being of non-Hispanic ethnicity and 70% were born in the United States. Most participants reported several years of schooling with 44% having at least a high school diploma or GED certificate, although 57% of all participants were unemployed at the time of the study. More than half (51%) of the participants reported never being married. Almost all of the participants (97%) reported ever testing for HIV. Fifteen participants (44%) reported testing in the last six months and additional descriptive analysis show that 11 of the women who had recently tested for HIV (i.e., in the last 6 months) were not married.

The qualitative findings below are described within the socioecological framework. Each domain, that is, HIV related knowledge, alcohol as a risk factor for HIV and HIV testing decision-making, is discussed within the framework’s levels of influence.

### 3.2. Individual Factors

Individual level factors such as beliefs, attitudes and knowledge can reduce women’s vulnerability to HIV and promote health seeking behaviors (e.g., HIV testing). Participants were knowledgeable about risk factors that contribute to the transmission of HIV such as injection drug use and high risk sexual behaviors (e.g., unprotected sexual intercourse, multiple sexual partners); and identified populations that have been disproportionately affected by HIV including people of color and men who have sex with men. These women underscored the importance of routine HIV testing, early diagnosis and adherence to ART. Participant’s HIV testing decision-making centered on enhancing knowledge and reducing the psychological stress of not knowing one’s HIV status. Participants also discussed their evolution of beliefs and attitudes about HIV transmission and treatment, and their perceptions of PLWHA. Participants recognized advances in HIV prevention including Pre-exposure prophylaxis (or PrEP) and HIV treatment, and discussed how HIV diagnosis today was no longer a death sentence, comparing it to one of many chronic illnesses:

“People are living a lot longer with it….When it first came out, people didn’t really know what to do…. As long as you are taking care of yourself, like if you have diabetes, you do what you are supposed to do. There is a possibility that you might be taken out by it [HIV/AIDS], just like any terminal illness that you have.”

[Urban Community—BK]

Despite vast knowledge about HIV risk factors, recently testing for HIV and understanding treatment for PLWHA, participants expressed fear of HIV infection and not feeling comfortable being around PLWHA. As this participant described:

“I know someone who has it and when they come to my house because they are close, almost like family, it is like, I lay out the plastic. Everything, cups, forks, spoons, I don’t want you eating off my dishes, I personally Clorox the toilet down. And I don’t mean to sound harsh. That’s just my feeling about it.”

[Urban Community—L]

Participants were also knowledgeable about the intersection of alcohol and HIV. Women discussed their own individual experiences and perspectives on the role of alcohol use as a driver for risk behavior and the effects of drinking on HIV transmission. Women described that alcohol use increased their susceptibility for HIV infection because alcohol tended to reduce inhibitions, putting them at greater risk for HIV risk behaviors. These women associated the consequences of alcohol use with poor judgment and poor decision-making, including poor condom compliance, encounters with multiple sexual partners and increased risk of sexual violence.

“If someone drinks alcohol, they lose their mind. They can [have] sex without knowledge, without mind, and anything can happen …. They are not thinking. Sometimes they don’t know that they are having sex.”

[Suburban Community—R]

Beyond describing the increased vulnerability and susceptibility for HIV infection due to alcohol consumption, participants discussed how alcohol use was a coping mechanism for some women to “overcome” and “escape” other problems such as inadequate financial resources, and emotional problems including anxiety, low self-esteem and lack of confidence. Moreover, participants reported that alcohol use helped them develop a tolerance for dealing with past and current unstable and abusive relationships.

“People try to cover a lot of emotional and mental stress using liquor because they don’t want to deal with it. They try and wash away the issues with liquor.”

[Suburban Community—C]

### 3.3. Interpersonal Factors

Interpersonal factors such as individual and group relationships, social networks, social supports, and social pressures and expectations can influence health behavior. Participants described relationships with their family, friends and social networks in relation to HIV knowledge, alcohol as a risk factor for HIV infection and HIV testing decision-making. Interpersonal relationships highly influenced HIV testing decision-making. Most women expressed that they tested for HIV to confirm their HIV status before beginning intimate relations with a partner. Participants viewed testing as a process for determining relationship viability. Knowing one’s HIV status and that of your partner was perceived as part of personal responsibility in the new relationship. Participants expressed that dual-partner testing was a key factor in building trust in a relationship; for instance, as the relationship became more serious, HIV testing facilitated the transition from protected to unprotected sexual encounters.

“Asking is no longer enough. When you are dating a guy, ask him by any means, “do you have the disease?” We cannot just ask whether the person has AIDS. It has to be official on a piece of paper. Let us go together [to get tested]. If the person cares about you, we go together and have the test. And that’s how you have to be because people are not going to be honest with you. At the end of the day, you are responsible for you. Nobody else.”

[Urban Community—BK]

Lastly, participants reported that testing was necessary because of prior relationship experiences. For example, women reported that they may have at one time or another engaged or had a present or former partner who may have been engaged in HIV risk behaviors such as having multiple sexual partners or unprotected sexual encounters.

At the interpersonal level, participants extensively discussed knowledge, attitudes and perceptions of HIV within the context of stigma. For these women, the social stigma of talking about HIV presented a layer of complexity in relationship building and sometimes reinforced behaviors that put women at risk for HIV infection. For instance, women reported anxiety and fear when discussing HIV with potential partners due to the stigma (internalized and structural) of being diagnosed with HIV. Participants worried about the negative judgments that the partner would have about them which discouraged women from disclosing their own HIV status and or inquiring about their partner’s HIV status. Participants discussed how inquiry of a partner’s HIV status, regardless of marital status, indicated distrust or infidelity. As this participant described:

“You know, if you are trying to get to know someone, you don’t want to ask that question [HIV status], and then suddenly you put a scarlet letter on yourself, and they are like, “oh my gosh, why is she asking me this”? and so people start questioning, “well, do you have it? have you been with someone who has it? why are you concerned about it?””

[Urban Community—M]

The fear and anxiety that these women have in talking about HIV with a partner could very well put these women at risk for HIV or impede efforts to build meaningful relationships.

Despite this delicate balance between protecting oneself from HIV infection and building a relationship with a partner, women expressed that HIV testing was imperative in relationship building, particularly for Black and African American women. This sentiment was echoed when women discussed building relationships in socially and economically disadvantaged communities that have higher rates of individuals re-entering the community following incarceration. While participants recognized the challenge of inquiring about a partner’s HIV status, women emphatically discussed how Black and African American women have the power to negotiate the sexual encounter by asking to see the results of a partner’s HIV test or having both partners testing for HIV simultaneously.

“You have to test. When you are going out with somebody, you don’t know that person so you can say, let’s go together to get tested.”

[Suburban Community—R]

In the context of HIV transmission, this interplay between interpersonal relationships and intimate relationship functioning plays a critical role in HIV protective behavior for Black and African American women.

When asked to describe the relationship between alcohol and HIV in the context of interpersonal factors, participants discussed how alcohol use helped them build confidence and reduce inhibitions when engaging with other people in social settings, and that alcohol use sometimes influenced sexual partnering. In exploring the relationship between alcohol use and HIV at the interpersonal level, the cluster of themes were similar to those described when women discussed the role of alcohol and HIV. Specially, women discussed how alcohol use reduced inhibitions when interacting with people in their social networks or meeting new people, resulting in the inability to make clear and conscious decisions and increased vulnerability and engagement in HIV risk behaviors.

### 3.4. Community Factors

HIV risk and protective behaviors are shaped by broader community contexts, i.e., formal or informal standards or norms among individuals, groups, and organizations that can hinder or facilitate health behavior change. Communities whether by identity (e.g., communities of color, women of color), relationships among organizations and groups (e.g., churches, agencies), geographic designation (urban, suburban, rural) or social networks (formal and informal) provide important power structures and organizational or group support partnership connections that can be leveraged to improve individual and population health. Across all groups, participants discussed the importance of multifaceted HIV prevention initiatives in their communities. Firstly, participants described the need for more HIV related information and education to increase knowledge with an emphasis on gender responsive approaches that incorporate women and girls’ empowerment strategies. Participants also discussed widening the reach for HIV prevention efforts across age groups (e.g., teens, youth, older adults) and in various settings (e.g., houses of worship, schools, libraries, workplace, neighborhood block meetings). As these participants described:

“We need to [have] informational sessions where older women can go for classes.”

[Suburban Community—C]

“A lot of people go to church and you don’t hear the church discussing more. They are bringing God. They need to do a little bit more and be out with HIV education because there are lot of women in the churches. And like I said, there is a lot of pride and stigma so if the church makes them comfortable enough to come to church, they should make it comfortable to talk about HIV. I don’t hear it and I don’t see much of it.”

[Suburban Community—S]

Secondly, participants also recognized that individual level behavior change involves a reciprocal relationship between the individual and community contexts, particularly community supports. A major theme across groups was the need to develop important skills for both children and their families. Participants described how community programs that enable women and girls to learn and develop actionable life skills such as conflict resolution and mediation, problem-solving, social and effective communication would be beneficial in negotiating and advocating for HIV protective behaviors in their interpersonal relationships. Within the context of alcohol use and HIV, participants described how education and life skills training such as self-esteem and knowing how to deal with peer pressure could reduce negative consequences associated with alcohol use.

“Alcohol is rampant in our communities.... It is an epidemic because education has been downplayed… there is not enough of us educating us about the disease and why we should stay on top of it.”

[Urban Community—B]

Participants expressed that there was a lack of family and community dialogue about HIV across and within generations of families, and this was a major barrier to addressing HIV related stigma across generations of families and hindered early HIV testing for Black and African American women. Participants suggested that in addition to providing multifaceted information and skill-based training, community agencies and organizations (e.g., health centers, religious institutions, YMCAs) should provide social safety nets for families to improve relations, strengthen knowledge and skills within families about health and foster HIV stigma reduction dialogue as part of community health improvement efforts.

“I think a lot of families don’t have the tools they need really to talk to their children. Even informational packets going out to the families, resources going to the families, media around it to show that this is happening in your community…… It has to be made known that this is happening to us and we are totally in control of stopping it.”

[Urban Community—BK]

“If you are not having any conversations, if you are not having knowledge being brought to the community, information being brought [to] the forefront, being brought to families, being brought to churches, being pushed in people’s faces so that they have to open their eyes to it. When that is not happening, how can you expect anyone to be comfortable with saying that I may not be having sex with anyone right now but I am going to go alone and get tested.”

[Urban Community—M]

Thirdly, participants discussed how the intersection of culture and gender influenced the nature of conversations in the family. Specifically, cultural norms and values determined if and how women could have a dialogue about sex and HIV prevention in the family. In addition, gender determined the extent/content/depth of conversation that the parent/caregiver had with their child. Participants described providing less or no specific information in mother–daughter dyads, illustrating important missed opportunities and the need to strengthen communication in mother–daughter dyads.

“I am guilty. Because of my culture and my country and where I work, we don’t talk about things like that. It is disrespectful to talk about it. When you get there, you will know. I have found that with my daughter, I said, you shouldn’t be doing that because mama says so. But we didn’t have the conversation about this is what is happening. With my son, it is a little different. I tell my son, this is what is going to happen.”

[Urban Community—BK]

To facilitate dialogue in community settings, participants discussed the importance of storytelling in leveraging dialogue in the community, because stories are akin to Black and African American heritage and the shared knowledge and message can transcend across church, beauty parlor, barbershop and other community settings.

Lastly, participants described that many HIV prevention programs in their communities were not culturally relevant to the contexts and lived experiences of Black and African American women, and that most did not extend beyond individual risk behavior. Participants expressed that there was an abundance of information and public campaigns for less stigmatizing illnesses such as cancer, diabetes and hypertension; and that unlike HIV public health messaging, these other public health communication campaigns tended to represent Black and African American women in a more favorable manner.

### 3.5. Societal Factors

Social and structural factors such as systems (justice, educational, healthcare), legislative and administrative supports (laws, policies, rules and regulations) and economic resources (poverty prevalence) can increase or reduce HIV risk exposure. Participants in this study discussed how the infrastructure of socially and economically disadvantaged communities increased women’s vulnerability to HIV transmission; and how the healthcare system influenced access to care, including HIV testing, care and treatment. Across all groups, participants were very knowledgeable about the fact that people of color, particularly Black and African American individuals, are disproportionately represented among persons with lower income, and therefore tend to reside in communities with high poverty prevalence and with less access to important social determinants of health. Women discussed how communities of color have fewer opportunities (e.g., economic, education, safe and affordable housing) and high disease burden (e.g., HIV/AIDS) as a function of past histories (e.g., residential segregation, discrimination) and community disinvestment. This generalized knowledge among Black and African American women reflects their shared historical and present experiences rooted in structural inequities.

When discussing the role of alcohol in HIV transmission, participants described how the availability of alcohol, i.e., alcohol outlet density, in communities of color increased accessibility, thus putting women at greater risk for alcohol-related risk behaviors. Participants discussed how inadequate alcohol zoning laws contribute to concentrated alcohol outlet density; and the lack of regulation of alcohol marketing and advertising in communities of color increased exposure and vulnerability to negative health consequences.

“Why do they take up every other goddamn corner with a liquor store?…. I hate the fact that in the inner city, every three or four blocks is a liquor store... I think we got like 12 liquor stores on this one street. Who needs 12 liquor stores on one street?…That’s sad. When you are in [town name omitted], you gotta drive four or five miles before you find one [liquor store]. Or you gotta walk a mile and by the time you get there and back you will be sober.

[Urban Community—B]

Participants discussed how the healthcare systems, including access to care and interactions with health care providers, influenced HIV related knowledge and HIV testing decision-making and practice.

Participants reported that primary care providers played an influential role in the promotion of HIV testing by discussing HIV risk behaviors with patients and offering those at risk voluntary HIV counseling and testing. However, most women expressed that having access to a primary care provider did not guarantee that one would get tested and that in most cases, getting tested depended on how proactive the patient or provider was in advocating for a HIV test. While many participants discussed that HIV testing should occur annually and as part of routine preventative care in a primary care or women’s wellness visit, most women reported that HIV testing was not readily discussed in a patient–provider interaction, and that often one had to specifically request a HIV test.

“When I go to the doctor I have a list of everything I want to get tested for. I write a list and say, this is what I want. You have to write a list. Some things you just have. And [for] HIV [testing] you just have to ask.”

[Suburban Community—S]

## 4. Discussion

Descriptive analyses illustrate that most study participants resided in urban communities, had a high school degree or equivalent, were unemployed and had tested for HIV in their lifetime. The socioecological framework recognizes that HIV risk and health seeking behaviors are shaped by the interactions that Black and African American women have with their environments. The nested levels of influence, i.e., individual, interpersonal, community and societal factors, interact to reduce or increase HIV infection exposure and vulnerability. The study findings show that there is a collective shared experience and that the intersection of all four levels of the socioecological framework is similar when examining HIV risk and protective factors among women residing in urban and suburban communities. The findings correspond to other studies that have examined: HIV related knowledge, attitudes and perceptions of HIV testing [86,87,88]; structural barriers and opportunities to HIV prevention [12,15,16]; and determinants of health associated with HIV acquisition [8,12,89] specifically among Black and African American women.

Results suggest that each level of the socioecological framework contributes to exposure and vulnerability to HIV infection. Women in this study identified determinants of HIV acquisition and transmission at the individual, interpersonal, community and social/structural level. At the beginning of each group, participants were invited to share their knowledge of the HIV disease. Findings show that these women had a wide breadth of knowledge and were able to describe the difference between HIV and AIDS, identify modes of HIV transmission, individual and structural factors that may contribute to HIV infection, populations disproportionately affected, testing methods including rapid HIV testing (oral, blood and urine testing) and advances in HIV prevention including PrEP and treatment. In their discussions, women sometimes shared information about where one could get specific resources (e.g., HIV testing clinics, gender specific support groups) in their communities, which suggests awareness of health resources and or engagement in health seeking behaviors.

Study findings show that fear, anxiety and the HIV stigma can impede HIV protective behaviors such as inquiring about a partner’s HIV status and disclosing one’s HIV status. These findings correspond to other research that has shown that HIV stigma plays an important role in how women go about seeking prevention and care services, disclosing HIV status, seeking family support, and ancillary services [25,26,27]. While almost all the study participants reported testing for HIV, it is important to recognize that stigma can have a negative effect on the health and health outcomes of those at risk and PLWHA. The psychological vulnerability associated with stigma may contribute to delaying or not seeking prevention and treatment services, thus putting women at greater risk of HIV infection and transmission. Studies show that HIV stigma is associated with HIV disclosure concerns [90], low social support [91], poor mental health including depression [90,91,92], lower HIV visit adherence [92,93], late initiation of ART [90] and reduced adherence to ART [90,92]. Evidence informed HIV prevention and treatment interventions must include stigma-reduction approaches with an emphasis on cultural relevancy and gender responsiveness.

Findings also show that engaging in community dialogue around HIV is an important avenue for promoting and leveraging HIV testing in communities of color. This is consistent with prior research that remarks the relevance of culture and gender specific HIV prevention initiatives for Black and African American women [94]. The literature also documents the need for more HIV prevention initiatives at the community level [94,95], specifically, community programs by and for women, which facilitate the development of comprehensive sexual health education programs, life skills based education, empowerment and resiliency building, serving as an essential community resource for women and girls. Programs by a collective of Black and African American women with shared experiences could enhance HIV prevention work around receptivity to and awareness of HIV, alcohol use and misuse and HIV testing.

Understanding health seeking behavior processes is central to best developing and implementing culturally relevant and gender responsive HIV prevention programming. Findings show that individual (HIV disease knowledge, attitudes, beliefs, perceptions) and interpersonal factors (past/current relationships, trust, relationship building) play a role in influencing why and when these women tested for HIV. Structural factors, particularly access to care, interactions with health care providers, self-advocacy during a patient-provider interaction and opt in–out HIV testing policies, also influenced how and when women got tested. While the Centers for Disease Control and Prevention (CDC) recommends routine testing, including non-risk based opt-out screening [96], study participants reported having to request a HIV test, thus suggesting that the CDC recommendations need to be better implemented by health care providers. Findings suggest that there is a need to train health care providers and establish standard clinical practice guidelines around HIV testing across multiple sectors to meet the needs of diverse populations. At a minimum, routine annual HIV testing in preventative primary care would ensure that undiagnosed HIV infections are identified in a timely manner. In addition, community public health efforts should ensure that community resources are available for adequate HIV testing beyond those provided in healthcare settings. Providing vast options for testing in either traditional healthcare or in community settings, and having health care providers, HIV testing counselors and community members engaged in the conversation, is imperative. These structural system changes would normalize and encourage widespread testing practices and perhaps reduce the social stigma associated with HIV.

There is a strong link between availability and accessibility of alcohol and negative health consequences in communities of color [97]. Findings demonstrate that women in this study are aware of the negative consequences associated with alcohol use, including the potential result of engaging in HIV risk behaviors while under the influence of alcohol. The role of alcohol as a risk factor for HIV infection is a complex phenomenon that goes beyond drinking behaviors. From the perspective of Black and African American women, results illustrate how structural inequities such as low economic resources in communities of color and inadequate legislative and administrative supports increase the availability of alcohol. Specifically, inadequate community infrastructure, alcohol zoning laws and aggressive alcohol marketing campaigns contribute to the disproportionate alcohol outlet density in communities of color. Reducing HIV incidence in communities with high HIV prevalence rates requires structural changes to promote investment in communities and decrease neighborhood disadvantage. Local and state policy makers should regulate and strengthen policies around the availability, accessibility, marketing and advertising of alcohol to mitigate alcohol-related risk behaviors in these communities. HIV prevention programming should also bolster interventions that raise awareness of the health and social problems caused by alcohol use and misuse.

A key contribution of this research is its use of the socioecological framework to systematically examine the role of alcohol use as a risk factor for HIV acquisition and transmission. While other studies have examined alcohol use and HIV testing among women [98], to the best of our knowledge, this is among the first studies to qualitatively examine perceptions of alcohol use as a risk factor for HIV acquisition and transmission for Black and African American women residing in urban and suburban communities. We explored this important question while also concurrently assessing HIV-related knowledge and HIV testing decision-making because protective factors such as HIV knowledge and HIV testing are central to mitigating HIV risk behaviors. Another contribution of this study is in its conceptualization using the PHCRP approach [70] and CRT principles [71] whereby our study focuses on exploring the diversity of attitudes, perceptions and experiences of Black and African American women in urban and suburban communities. The study also recognizes the intersection of race and gender as fundamental drivers of health for Black and African American women. Race and gender are inextricably linked to structural inequities such as economic inequity (e.g., overrepresentation in low-paying jobs, gender pay gap) [4], discrimination and marginalization [99]; thus, the social production of HIV in Black and African American women must be carefully understood and remedied. The socioecological framework requires multilevel and multifaceted interventions to reduce HIV risk and increase health seeking behaviors for Black and African American women. This study highlights the multi-dimensional factors that Black and African American women face in the context of HIV related knowledge, and HIV testing decision-making and perceptions of alcohol use as a risk factor for HIV. Evidence informed interventions to reduce the disproportionate burden of HIV among Black and African American women must recognize that HIV infections are not a product of behavior alone, and must address the interrelationship between individual, social and structural contextual factors that influence HIV acquisition and transmission. These interventions must be gender responsive and emphasize cultural relevancy and humility.

### Study Limitations

Participants in this study reside in urban and suburban communities from one state and therefore findings may not be generalizable. As with many qualitative studies, our study used purposive sampling. As such, study findings are not generalizable and may not reflect those of Black and African American women in other non-urban or -suburban communities in the U.S. We also acknowledge that there is bias introduced when participants self-select into a study. Possible selection bias was explored by analyzing demographic data from focus group participants to better understand how focus group participants differed. As noted in our results, there were no notable variations across and within groups. Participants who enrolled may have been interested in the topic and might have been more knowledgeable about HIV/AIDS. Self-report data about HIV testing status and duration since last HIV test were not verified using medical records and could be prone to recall bias. Our study did not ask participants to disclose the complex ancestry of their Black and African American racial identities, levels of alcohol use and HIV risk behaviors. Additionally, this study did not assess other HIV risk factors and multimorbidity, e.g., injection drug use and its relationship with alcohol use. These types of data would have enhanced within group and subgroup analyses. Future studies should collect much more nuanced data in order to better inform intervention tailoring for different subgroups of women. Despite these limitations, the strengths of this study include the use of a rigorous qualitative methodology that allowed for in-depth data collection and analysis, grounding this research in important public health frameworks and approaches, and an examination of concurrent risk and protective factors.

## 5. Conclusions

This study provides new and valuable information about HIV knowledge, HIV testing decision-making and perceptions of alcohol use as a risk factor for HIV for Black and African American women residing in urban and suburban communities. Study findings suggest that there is a need to develop and scale up HIV prevention programming that is culturally relevant and gender responsive; integrates knowledge building and life skills (e.g., problem solving, self-esteem, nurturing, mother–daughter dyad communication); and spans the age developmental spectrum. HIV prevention programming should be disseminated in traditional and non-traditional settings such as school networks, community centers, neighborhood block meetings and the social fabric of religious organizations.

Community investment development efforts that increase economic opportunities and community engagement are essential in communities of color. Building community capacity (e.g., organizational resources, social capital, community infrastructure), and the collective power and agency of people in those communities, can promote individual and population health. Reducing alcohol outlet density, regulating the distribution and marketing of alcohol in order to mitigate the negative consequences of alcohol use and restoring communities with evidence informed initiatives can enhance community investment and improve health.

Finally, training on HIV testing recommendations for primary and specialty care providers has the potential to increase the frequency of HIV testing without the added burden of a patient having to make a request. Ongoing patient–provider conversations about HIV can improve HIV knowledge, reduce misconceptions about HIV and move the needle on reducing HIV stigma. Routine testing would have tremendous impact by identifying HIV early in the disease progression, linking newly diagnosed individuals to appropriate medical care and social supports and attaining CDC’s goal of testing non-risk based opt-out screening.

## Figures and Tables

**Table 1:** Deductive and inductive approach to thematic analysis using the socioecological framework.

Conceptual Framework	Research Domain	Primary Category	Subcategory	Concepts Arising from Data
Individual/ Intrapersonal factors	Knowledge, beliefs, attitudes and perceptions of HIV/AIDS	HIV related knowledge	Infection, transmission, diagnose, symptoms, treatment	Definition of HIV/AIDS, HIV risk behaviors, concerns, HIV testing, non-testing, forms of care
		Attitudes and perceptions	Beliefs, perceptions of risk	Belief systems, religion, government policies
	Perceptions of alcohol use as a risk factor for HIV infection	Drinking behaviors	Social drinking, heavy drinking, perception of risk	Mental health (depression), stress, gateway to other substances, religion, sexual violence
		Attitudes and perceptions	Perceptions of risk	Social norms, gender roles
Interpersonal factors	Perceptions of alcohol use as a risk factor for HIV infection	Risk	Unprotected sexual contact, sexual violence, negotiation	Condom compliance, decision-making, inhibition, formal and informal networks/friends, relapse
	HIV testing decision-making	Relationships	Interpersonal relationships, HIV risky behavior engagement, diagnoses, disclosure	Concerns, gender roles, formal and informal networks/friends, forms of care, sexual identity, infidelity
Community factors	Knowledge, beliefs, attitudes and perceptions of HIV/AIDS	Historical and Cultural context	Historical context, gender roles, resource allocation community factors (poverty), discrimination, oppression	Cultural norms, disenfranchised communities, mistrust of research and government arenas
	Perceptions of alcohol use as a risk factor for HIV infection	Cultural context	Historical context, community factors (poverty), political and economic power, community inequities	Disenfranchised communities, social policies
	HIV testing decision-making	Cultural context	Historical beliefs, cultural biases, access to services, resource allocation	Clinical practice, attitudes and practices, access
Societal factors	HIV testing decision-making	Frequency of testing	Attitudes, access, knowledge	Provider relationship, forms of care, cultural norms, communities, media (Oprah, Dr. Phil), target groups
		Service delivery	Health promotion, clinical practice, resource allocation	Access, education, HIV testing policy guidelines, role of school systems, role of community based organizations

**Table 2:** Sociodemographic characteristics of Black and African American women (*n* = 37) ^a,b^.

Characteristic	*n* (%) or Mean (SD)
Community	
Urban	22 (59.5%)
Suburban	15 (40.5%)
Age	39 (SD 8.15)
Ethnicity	
Non-Hispanic	36 (97.3%)
Hispanic	1 (2.7%)
Country of origin	
U.S. born	26 (70.3%)
Non-U.S. born	11 (29.7%)
Education	
Less than high school	7 (19.4%)
High school graduate (High school diploma or GED certificate)	16 (44.4%)
Some college or more	13 (36.1%)
Employment status	
Employed	16 (43.2%)
Not employed—unable to work	4 (10.8%)
Not employed—student	2 (5.4%)
Not employed—retired	2 (5.4%)
Not employed—housemaker	2 (5.4%)
Not employed—other	11 (29.7%)
Marital status	
Currently married	4 (11.4%)
Widowed	1 (2.9%)
Divorced	9 (25.7%)
Separated	3 (8.6%)
Never married	18 (51.4%)
Ever tested for HIV	
Yes	34 (97.1%)
No	1 (2.9%)
Last time tested for HIV	
Less than 6 months ^c^	15 (44.1%)
More than 6 months, but not more than 1 year ago	6 (17.6%)
1 year ago or more	13 (38.2%)

^a^ Data were obtained from seven focus groups. Average number of participants 5 (SD 2.4). ^b^ Data may not add up to 37 due to missing data. ^c^ Of the 15 participants, 11 were not married.

## Data Availability

Data are not publicly available due to the confidentiality assured to study participants for their responses and participation in the study.
